# Retraction: AQP4-Dependent Water Transport Plays a Functional Role in Exercise-Induced Skeletal Muscle Adaptations

**DOI:** 10.1371/journal.pone.0345808

**Published:** 2026-03-26

**Authors:** The PLOS One Editors

After the publication of this article [1,2], concerns were raised about Figs 1, 2*, 4*, 8*, and S1. Specifically,

- Multiple similarities were noted between lanes within the following panels:
  - Fig 1B EDL and QUAD panels.
  - Fig 1E.
  - Fig 2A*.
  - Fig 4A* Wheel Running panel.
- When levels are adjusted to visualize the background, there appear to be multiple vertical discontinuities in the background of the following panels:
  - Fig 1B QUAD panel.
  - Fig 2A*.
  - Fig 4A* Wheel Running panel.
  - Similarities were noted between multiple regions in:
  - Fig S1A EDL and SOL, within and between panels.
  - Fig S1A FDB panel.
  - Fig 8E* β-DG panel.

The corresponding author responded to the above concerns and confirmed that the original underlying data are no longer available. In the absence of the original underlying data, the image concerns cannot be resolved.

In light of the above unresolved concerns, which question the integrity and reliability of the reported results and conclusions, the *PLOS One* Editors retract this article.

FP, GPN, MS, and AF agreed with the retraction. DB, BB, AS, GPN, MGM, and CR either did not respond directly or could not be reached.

*The numbering of these figures was updated during a previous correction [2] and this notice refers to the corrected figure numbers.
